# About Surgery, Ketamine, and Quality Improvement

**DOI:** 10.31486/toj.22.5028

**Published:** 2022

**Authors:** Ronald G. Amedee

**Affiliations:** Director of Clinical School and Professor, The University of Queensland Medical School, Ochsner Clinical School, New Orleans, LA; Editor-in-Chief, *Ochsner Journal*

*Alas, summer sun can’t last forever. The days will grow cooler and shorter, and our skin will once again pale.*

*–Sarah MacLean*

The Fall 2022 issue of the *Ochsner Journal* contains four original research articles, a quarterly column, two quality improvement articles, and nine case reports and clinical observations which serve as the principal elements for this edition. Surgery—orthopedics, otolaryngology, and oncology—is well represented in this issue; we have reports of two different uses of ketamine; and we have an example of a quality improvement project that keeps on improving.

Concerned about the high mortality figures being published for patients undergoing orthopedic surgery during the COVID-19 pandemic, our colleagues at LSU-Shreveport decided to look at the outcomes of their orthopedic trauma patients. As the paper title states, “Low Mortality of Orthopedic Trauma Patients with Asymptomatic COVID-19: A Level I Trauma Center Pandemic Experience,” their outcomes were extremely reassuring.

Our friends at LSU-Baton Rouge continue to assess the results of the 2016 Medicaid expansion in Louisiana and the impact from the work of the Franciscan Missionaries of Our Lady (FMOL) Health System. To address the critical health care access problems resulting from the closure of the Earl K. Long Medical Center, FMOL opened both an urgent care center and a freestanding emergency department in north Baton Rouge. “Impact on an Urgent Care Clinic of a New Freestanding Emergency Department in a Resource-Scarce Area” details the results.

In the cosmetic surgery arena, “Preserving Nasal Tip Rotation and Projection in Open Septorhinoplasty” not only reports the results of three procedures but also gives us this issue's cover art by talented Ochsner Medical Illustrator Barbara Siede.

Two articles in this edition deal with the use of ketamine. Khatib, Roelofsz, Singh, et al examine “Hemodynamic Effects of Ketamine Infusion in the Intensive Care Unit for Maintenance Sedation Compared With Propofol and Midazolam: A Retrospective Cohort Study.” The sample sizes for this study are small, but the results can potentially be used in a future meta-analysis to provide a more complete picture of the hemodynamics of these three sedation medications. The other ketamine-related paper is a case report entitled “Ketamine as an Analgesic Adjunct for Opioid-Induced Hyperalgesia in a Patient With a Sickle Cell Pain Episode.” In an interesting clinical scenario, low-dose ketamine was used to help wean a patient off an opioid infusion.

Our Orthopedic Sports Medicine quarterly column, authored by Caballero, Kobayashi, and Gottschalk, is written with primary care physicians in mind and focuses on choosing the appropriate local anesthetic to use with musculoskeletal injections.

In the quality improvement realm, “Improving Operating Room Efficiency: Relocating a Surgical Oncology Program Within a Health Care System” provides a detailed look at the logistics of moving an established surgical program from one hospital to another. Orr, Thompson, Bruens, et al provide extensive drill-down metrics from before and after the move that highlight areas for potential additional efficiencies that aren’t immediately obvious from the high-level metrics.

“Improving Pneumococcal Vaccination Rates in an Inpatient Pediatric Diabetic Population” by Mirza, Jagadish, Trimble, et al is a great example of “you don’t know what you don’t know.” The authors designed an intervention to improve vaccination ordering, and the intervention was successful. But the authors discovered that ordering a vaccine didn’t ensure the vaccine would be administered. In a great example of true quality improvement, they identified a problem they didn’t know existed, shifted course, and developed potential solutions.

Case reports in this issue include two papers from Ochsner Maternal-Fetal Medicine, one about a mom and one about a baby: “Atropine, Ondansetron, and Ketorolac: Supplemental Management of Amniotic Fluid Embolism” and “Accidental Ingestion of a NEO-fit Device Component by a Neonate.”

I’m preparing this introduction in mid-August. The remainder of this month and the one to follow typically determine our tropical weather fate in the Gulf South. We cannot afford to let our guard down at this time, and we have learned to expect the unexpected when it comes to weather events in our area. However, the Ochsner Health executive team is quite experienced in crisis leadership, and our system has a history of demonstrating organizational resiliency both during and in the aftermath of hurricanes. Let us hope and pray that we do not need to demonstrate either of these qualities during the remainder of this calendar year.

